# Modulating the Effect of β-Sitosterol Conjugated with Magnetic Nanocarriers to Inhibit EGFR and Met Receptor Cross Talk

**DOI:** 10.3390/pharmaceutics15082158

**Published:** 2023-08-19

**Authors:** Shanmuga Sundari Ilangovan, Biswanath Mahanty, Venkatesan Perumal, Shampa Sen

**Affiliations:** 1 Department of Biotechnology, Bannari Amman Institute of Technology, Sathyamangalam 638401, India; 2 Division of Biotechnology, Karunya Institute of Technology and Sciences, Coimbatore 641114, India; bmahanty@karunya.edu; 3 Center for Injury Biomechanics, Materials and Medicine, Department of Biomedical Engineering, New Jersey Institute of Technology, Newark, NJ 07102, USA; perumal@njit.edu; 4 School of Biosciences and Technology, Vellore Institute of Technology, Vellore 632014, India

**Keywords:** β-sitosterol, EGFR, MET, SPIONs, PEG, PNIPAM, drug adsorption, drug release, cancer

## Abstract

The cross-talk between the EGFR (Epidermal Growth Factor Receptor) and MET (Hepatocyte Growth Factor Receptor) poses a significant challenge in the field of molecular signaling. Their intricate interplay leads to dysregulation and contributes to cancer progression and therapeutic resistance. β-Sitosterol (BS), a plant sterol with promising anticancer properties, shows increased research on its potential as a chemopreventive agent. However, significant modifications are required to deliver BS in cancer cells due to its lower efficacy. The present work aims to design a carrier-mediated delivery system specifically targeting cancer cells with EGFR and MET receptor cross-talk. Surface modification of BS was performed with superparamagnetic iron oxide nanoparticles (SPIONs), polyethylene glycol (PEG), and poly(N-isopropylacrylamide) (PNIPAM) to enhance the delivery of BS at the target site. BS was conjugated with SPIONs (BS-S), PNIPAM (BS-SP), PEG, and PNIPAM (BS-SPP) polymers, respectively, and the conjugated complexes were characterized. Results showed an increase in size, stability, and monodispersity in the following order, BS-S, BS-SP, and BS-SPP. The drug encapsulation efficiency was observed to be highest in BS-SPP (82.5%), compared to BS-S (61%) and BS-SP (74.9%). Sustained drug release was achieved in both BS-SP (82.6%) and BS-SPP (83%). The IC 50 value of BS, BS-S, BS-SP, and BS-SPP towards MCF 7 was 242 µg/mL,197 µg/mL, 168 µg/mL, and 149 µg/mL, HEPG2 was 274 µg/mL, 261 µg/mL, 233 µg/mL and 207 µg/mL and NCIH 460 was 191 µg/mL, 185 µg/mL, 175 and 164 µg/mL, indicating highest inhibition towards NCIH 460 cells. Our results conclude that β-sitosterol conjugated with SPION, PEG, and PNIPAM could be a potential targeted therapy in inhibiting EGFR and MET receptor-expressing cancer cells.

## 1. Introduction

β-Sitosterol (BS) is a prominent phytosterol that is found abundantly in plants and vegetables. It possesses remarkable bioactivity, including notable anti-inflammatory, antibacterial, and antifungal properties [1]. BS is a natural nutritional supplement that has been extensively studied and proven safe, offering numerous health benefits. It effectively regulates pain levels and inflammation by modulating the production of inflammatory cytokines, thereby assisting in the management of allergies and prostate enlargement. Furthermore, BS exhibits potent anticancer activities by enhancing natural killer cell activity and promoting the production of immune cytokines, effectively inhibiting tumor metastasis [2]. It also triggers apoptosis in leukemia cells through various cellular mechanisms. By affecting multiple cell signaling pathways, this bioactive phytosterol induces apoptosis and inhibits cancer cell proliferation, invasion, survival, and angiogenesis [2,3,4]. BS shows promising anticancer effects in colorectal cancer by inhibiting the EGFR/AKT signaling pathway [5]. In our previous work, we found that BS had a strong binding affinity towards both EGFR and MET receptors [6]. BS is naturally a hydrophobic molecule with a chemical structure similar to cholesterol. Due to its higher hydrophobicity and low water solubility, the efficacy of BS remains lower. This limits the effectiveness of BS in drug delivery and other applications. Despite its significant advantages, BS is often considered an “orphan phytosterol” [1], indicating its underutilized potential in various fields.

The bioactivity of BS can be improved by conjugating it with carrier molecules. SPIONs, which are superparamagnetic iron oxide nanoparticles, are utilized as carriers for targeted drug delivery in various therapeutic applications. These nanoparticles possess biocompatibility, and the residual iron content is metabolically utilized by the human body. SPIONs accumulate in the kidney for a short period of time and are excreted. Another possible way of excretion of SPIONs is by exocytosis [7,8]. Due to their excellent physicochemical properties [5], SPIONs have gained significant recognition among scientists, particularly in the field of cancer therapeutics and in the treatment of diverse diseases. The high surface-to-volume ratio of SPIONs allows for efficient drug loading and transportation. Decreasing the particle size to less than 100 nm increases bioavailability and leverages the enhanced permeability and retention (EPR) effect [9,10]. Moreover, SPIONs facilitate the accumulation of drugs at the desired target site.

SPIONs possess distinctive physicochemical characteristics that make SPIONs highly suitable for various applications, especially in drug delivery systems. Some of their key advantages include controllable surface charge, stable colloidal properties, inherent magnetic behavior, biodegradability, and biocompatibility. Moreover, SPIONs maintain their structural integrity during transportation and delivery, ensuring the drugs they carry remain intact until reaching the target site [11]. Unlike liposomal and lipid-based nanoparticles (LNPs), SPIONs are stable for longer periods and can stay at the target location for an extended period, allowing for sustained drug release [12] while also being efficiently removed from the body later on. Since SPIONs are typically hydrophobic in their pure form, hydrophilic coatings on SPIONs with polymers allow them to become more water-soluble or form stable colloidal suspensions in aqueous solutions. These surface modifications also help prevent agglomeration and enhance the stability of SPIONs in physiological environments.

However, common issues observed with SPIONs are instability, agglomeration, and rapid drug release. RES uptake is a common problem, causing entrapment in the liver or lungs and increasing nanoparticle toxicity. To address these challenges, surface coating of SPIONs is necessary. Biodegradable polymers such as polyethylene glycol (PEG) and poly(n-isopropylacrylamide) (PNIPAM) are commonly used for stabilizing SPIONs in drug delivery applications [10,13]. PEG reduces RES uptake and prevents agglomeration [14], while PNIPAM is a thermo-responsive polymer that undergoes a phase transition at body temperature [15]. These polymers have been successfully integrated with SPIONs to create composite hydrogels for drug delivery. Hence, we explored the use of these hydrophilic, biodegradable polymers in achieving sustained drug release and controlled temperature conditions for efficient drug delivery.

In the current investigation, we focus on enhancing the drug delivery efficiency of BS, a compound exhibiting high affinity towards both EGFR and MET receptors and known to inhibit cross-talk between these receptors in non-small cell lung cancer (NSCLC). To achieve this objective, we employed the conjugation of BS with SPIONs. Further, to improve their efficiency, SPIONs and BS were conjugated with PEG-PNIPAM polymers. We aim to augment the therapeutic potential of BS by enabling precise localization of the compound, potentially leading to improved treatment outcomes while minimizing adverse effects on non-target tissues.

## 2. Materials and Methods

### 2.1. Chemicals, Reagents, and Cell Lines

Fetal bovine serum was obtained from Sigma Aldrich. All the solvents, chemicals, and reagents were purchased from Hi Media laboratories (Mumbai). Cancer cell lines viz., MCF 7 (human breast cancer), HEP G2 (liver cancer), NCIH 460 (non-small cell lung cancer), and non-cancerous human kidney cell line, HEK293 were procured from the National Centre for Cell Sciences (NCCS), Pune.

### 2.2. Synthesis of β-Sitosterol Conjugated SPIONS

In our previous study, [6] we explained the synthesis of SPIONs by the co-precipitation method. Specifically, we employed the co-precipitation of 0.1 M FeCl_3_ and FeSO_4_ salts. For the preparation of BS-conjugated SPIONs, we modified the co-precipitation method originally described by Silva et al. in 2013 [16]. Initially, 100 mg of dried SPIONs was dispersed in 50 mL of ethanol and sonicated for 20 min to achieve uniform dispersion. Subsequently, a solution containing 50 mg of BS dissolved in a mixture of 50 mL ethanol and chloroform in a 2:3 ratio was added slowly to the SPION dispersion while maintaining constant stirring at 400 rpm on a magnetic stir plate. The resulting mixture was stirred at 400 rpm for 24 h at room temperature to develop uniform BS-conjugated SPIONs (referred to as BS-S). After 24 h, the solvent was evaporated at room temperature to obtain the BS-SPIONs conjugated complex.

### 2.3. Preparation of BS Conjugated SPIONs- PNIPAM (BS-SP) Polymeric Complex

SPIONs were conjugated with PNIPAM-NH_2_ terminated polymer with slight modifications [17]. Then, 0.25 g of the dried SPIONs was dispersed in 10 mL of ethanol and sonicated for 2 h to achieve a well-dispersed solution. Subsequently, an equal amount of PNIPAM-NH_2_ (0.25 g in 10 mL ethanol) was added to the SPIONs, stirred at 800 rpm at 28 °C for 12 h. This step facilitates the conjugation of the amino groups with the SPION’s surface [17]. The resulting polymeric-coated SPIONs (SP) were washed three times with ethanol and dried to remove any unreacted polymers and impurities.

The subsequent conjugation of β-sitosterol’s carboxylic acid group with the amine terminus of PNIPAM-SPIONs employed an EDS/NHS coupling mechanism. In brief, 1.18 g of 1-Ethyl-3-(3-dimethylaminopropyl) carbodiimide (EDC) and 0.68 g of N-hydroxysuccinimide (NHS) were dissolved in 50 mL of ethanol. The entire content was mixed with β-sitosterol (0.5 g) and SPIONs conjugated PNIPAM (0.5 g). The reaction was carried out with magnetic stirring for 12 h at 28 °C. The reaction mixture was washed thrice with ethanol to remove unreacted materials [18]. The entire content was dried at room temperature to get BS-conjugated SPIONs-PNIPAM (BS-SP).

### 2.4. Preparation of BS Conjugated SPIONs-PEG- PNIPAM (BS-SPP)

In order to conjugate the carboxylic acid group of BS with the amine terminus of PNIPAM, an EDS/NHS coupling mechanism was employed (as described above). In brief, 1.18 g of EDC and 0.68 g of NHS were dissolved in 50 mL of ethanol. Subsequently, equal amounts of BS and PNIPAM were added to the solution. The reaction was carried out under magnetic stirring for 12 h at 28 °C. To remove unreacted polymers and impurities, the reaction mixture was washed three times with ethanol and dried, resulting in the formation of BS-conjugated PNIPAM (BS-PNIPAM). Simultaneously, the SPIONs were combined with an equal amount of PEG (polyethylene glycol) and subjected to 12 h of stirring. The resulting SPIONs conjugated with PEG (SP-P) were washed to eliminate any unreacted polymers and dried [18]. Finally, the SP-P was mixed with the BS-PNIPAM complex in a 1:1 ratio and stirred for 12 h. The resulting mixture was washed five times with ethanol and dried, yielding BS-conjugated SPIONs-PEG-PNIPAM (BS-SPP). The obtained BS-SPP are stably redispersed in ethanol.

### 2.5. Characterization of SPIONs, BS-S, BS-SP, BS-SPP

The morphological characteristics of SPIONs, BS-S, BS-SP, and BS-SPP were analyzed using various analytical techniques. The particle size, size distribution, and zeta potential of the synthesized nanoparticles were investigated with a Nanopartica SZ-100 instrument (Horiba Ltd., Kyoto, Japan) based on the dynamic light scattering (DLS) technique [19,20,21]. To perform the analysis, 30 µL of a 1 mg/mL suspension of SPIONs, BS-S, BS-SP, and BS-SPP were added to 5 mL of distilled water and ultrasonicated for 20 s. Readings were taken, and three runs were conducted for each sample to obtain an average reading. The size of the nanoparticles was examined using transmission electron microscopy (TEM, Jeol JEM 2100, SAIF, Cochin) [20,21]. For this analysis, each sample with a concentration of 500 µg/mL in water was ultrasonicated for 20 s. Subsequently, 50–100 µL of the prepared sample was placed on a copper TEM grid. The excess sample was carefully removed using filter paper, and the remaining sample was air-dried before observation under the TEM.

### 2.6. In Vitro Adsorption of β-Sitosterol onto SPIONs

The drug loading characteristics of BS onto SPIONs, SPIONs-PEG, and SPIONs-PEG-PNIPAM were studied as follows: 2 mg of the prepared BS-S, BS-SP, and BS-SPP were mixed with 20 mL of ethanol. The solution was placed in a shaker maintained at 100 rpm at 28 °C. Every 1 h, 2 mL sample was extracted from the solution in the presence of an external magnet, and the removed volume was replaced with 2 mL of fresh ethanol. The adsorption of the drug was quantitatively analyzed with a UV-Vis spectrophotometer at 206 nm [22,23]. The drug encapsulation efficiency, or the amount of drug adsorbed, was calculated using the equation given below (1).

(1) $$Drug\;encapsulation\;efficiency=\frac{Total\;amount\;of\;drug-free\;drug\;in\;the\;supernatant}{Total\;amount\;of\;drug}\times 100$$

### 2.7. In Vitro Release of β-Sitosterol from SPIONs

The drug-release behavior of BS was investigated using a dialysis method [24]. In this study, 2 mg of each BS-S, BS-SP, and BS-SPP was dispersed in 2 mL of phosphate buffer saline (PBS) and placed in a dialysis bag. The bag containing the BS-S dispersion was immersed in a beaker filled with 200 mL of PBS and stirred at a rate of 100 rpm. Regularly, at specific time intervals, 2 mL of the sample was withdrawn from the dialysis bag and replaced with 2 mL of fresh PBS solution. The amount of drug released was determined spectrophotometrically at 206 nm, enabling the evaluation of the cumulative release behavior of BS from the SPIONs over time.

### 2.8. Cell Viability Assay

The impact of SPIONs and BS-S, BS-SP, and BS-SPP on cancer cell viability was assessed with an MTT dye reduction assay [25]. Butein, a dual inhibitor [26] of the EGFR and MET receptors, was used as a positive control. HEPG2, MCF7, NCIH460, and HEK293, were seeded in 96-well tissue culture plates. Each well contained cells with a concentration of 1 × 10^6^ cells and the plates were incubated for 48 h at 37 °C in a CO_2_ incubator. SPIONs, BS-S, BS-SP, BS-SPP, and Butein, were suspended in DMSO (dimethyl sulfoxide) and diluted with the cell culture medium at various dosages (500, 250, 125, and 62.5 mg/mL). The cells in the 96-well plates were exposed to different concentrations of SPIONs, BS-S, BS-SP, BS-SPP, and Butein, followed by further incubation. After incubation, the supernatant was discarded, and 200 μL of MTT (5 mg/mL) was added to each well, followed by a 4 h incubation. The resulting formazan crystals were dissolved by adding 100 μL of DMSO. DMSO-treated cells served as the background control, while untreated cells acted as the negative control. The optical density of the cells was measured at a wavelength of 540 nm. The relative cell viability was calculated as the absorbance ratio of the test sample to that of the control, multiplied by 100.

### 2.9. Flow Cytometric Analysis

Flow cytometric analysis was conducted to evaluate the expression of EGFR and MET receptor proteins in the NSCLC cell line NCIH460 [27]. Cells were cultured in a 6-well plate at a density of 3 × 10^5^ cells/2 mL and were incubated in a CO2 incubator at 37 °C for 24 h. Samples, viz., BS, BS-SP, BS-SPP, and the control drug, berberine [28], at their effective concentrations determined from MTT analysis, were added to 2 mL of culture medium and were incubated for 24 h. After incubation, the media was removed, and cells were washed with PBS. Subsequently, Trypsin-EDTA solution (200 μL) was added to the PBS-washed cells, and they were incubated at 37 °C for 3–4 min. The cells were harvested directly into 12 × 75 mm polystyrene tubes. The cells were washed with PBS and reconstituted with anti-EGFR FITC antibody according to the manufacturer’s instructions, and 5 µg/mL of antibody was added to the cells and incubated for 30 min in the dark. The cells were washed twice with PBS, re-suspended in 500 μL PBS and mixed well prior to analysis. Flow cytometric analysis was conducted using the 488 nm laser for excitation and 535 nm for detection.

A similar protocol was employed to evaluate MET receptor expression with slight modifications to the above method with quercetin [29] as a control. After Trypsin-EDTA incubation, the cells were washed twice with PBS, fixed with 80% methanol (5 min), and permeabilized with 0.1% PBS Tween for 20 min. The cells were then incubated in 1X PBS/with 0.5% bovine serum albumin (BSA) to block non-specific protein–protein interactions, followed by the antibody (ab51067, 1/1000 dilution) for 30 min at 22 °C. The cells were then washed with 1X PBS and 0.2 mL (5 µg/mL) of goat anti-rabbit IgG (H + L) secondary antibody, FITC, and incubated in the dark for 60 min at RT. Prior to analysis, the cells were washed with PBS and mixed thoroughly with 500 μL of PBS.

### 2.10. Statistical Analysis

Statistical calculations were conducted using GraphPad Prism software (9.4.1). The data are reported as the mean ± standard error of the mean (SEM). Significance levels were determined based on *p*-values, with values below 0.05 (*) considered statistically significant.

## 3. Results

### 3.1. Synthesis and Characterization of BS-S, BS-SP, BS-SPP Conjugated Complexes

The β-sitosterol was successfully conjugated with SPIONs, SPIONS-PNIPAM, and SPIONS-PEG-PNIPAM. Each conjugated complex was subjected to characterization studies and evaluated for its maximum drug adsorption and sustained drug release properties.

#### 3.1.1. Hydrodynamic Size and Stability Studies of BS-S, BS-SP and BS-SPP

**Figure 1:**
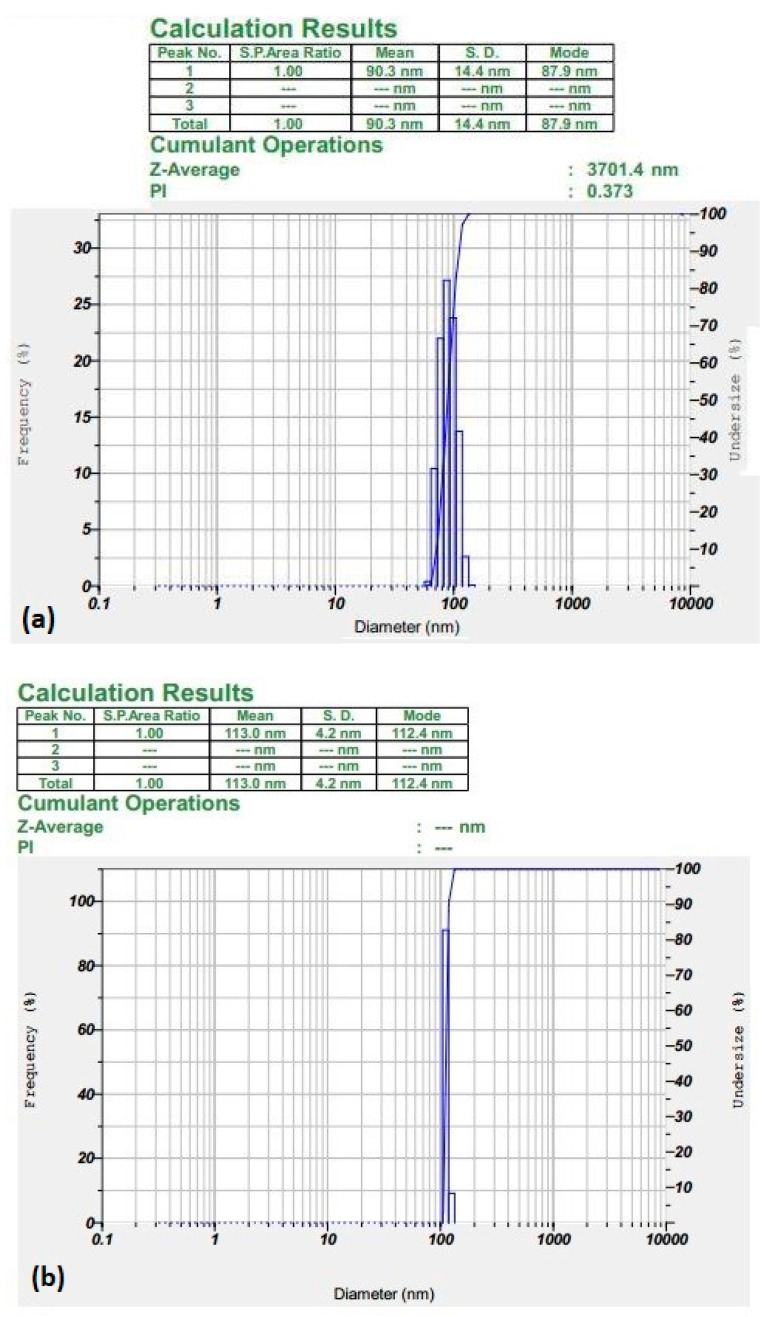

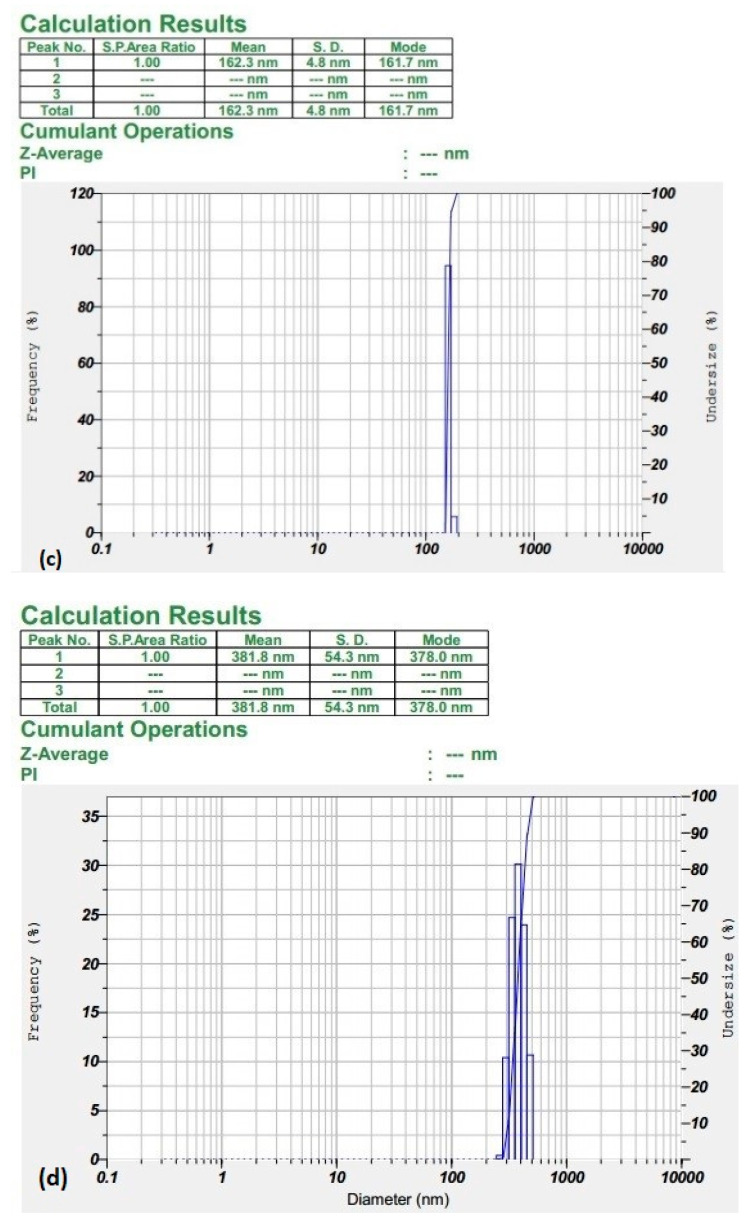
(**a**,**b**) Represents the hydrodynamic size of SPIONs and BS-S. (**c**,**d**) represents the hydrodynamic size of BS-SP and BS-SPP.

The results of the mean hydrodynamic size and zeta potential measurements for SPIONs and polymer conjugates are summarized in Table 1. In particular, the size of SPIONs was found to be 90.3 nm, as shown in Figure 1a. This was comparatively smaller to BS conjugated SPIONs which had a mean hydrodynamic size of 113 nm, as depicted in Figure 1b. These conjugated SPIONs demonstrated a narrow size distribution with monodispersed particles. The observed increase in size for the BS-conjugated SPIONs indicates the successful conjugation of the drug with the SPIONs. Further, the addition of PNIPAM-NH2 increased the size of BS-S from 113 nm to 162 nm in the BS-SP complex (Figure 1c). To further enhance the stability and drug absorption/release characteristics of BS-S, PEG was added together with the PNIPAM-NH2 polymer. The addition of two polymers drastically increased the size of the complex to 381 nm, as shown in Figure 1d.

Further, an increase in stability was achieved with SPION-conjugated polymers through zeta potential analysis. SPIONs showed a zeta potential of −15.8 mV (Figure S1a, whereas BS-S displayed higher stability with a zeta potential of −48.7 mV (Figure S1b). This significant increase in stability suggests that the conjugated complex is more resistant to aggregation or sedimentation [30]. From Figure S1c and Figure 1d, it was evident that higher stability was achieved with the BS-SP (−54.2 mV) and BS-SPP (−62.4 mV) complex, indicating the increase in stability due to the addition of polymer. The addition of a polymeric agent could naturally increase the stability of the nanoparticle drug complex.

#### 3.1.2. TEM and SEM Analysis of BS-S, BS-SP, and BS-SPP

**Figure 2:**
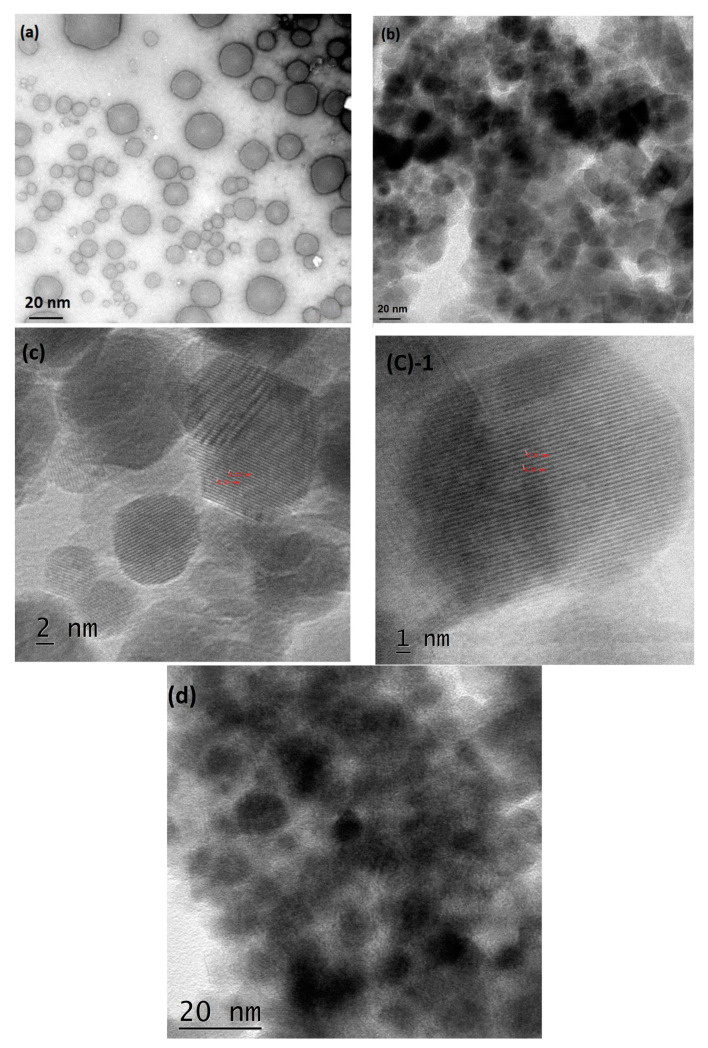
Structural elucidation of beta sitosterol conjugated with SPIONs and polymers. (**a**–**d**) represent the size of BS-S, BS-SP, BS-SPP, and SPIONs observed through TEM analysis.

The TEM analysis of BS-S, as illustrated in Figure 2a, demonstrated the presence of monodispersed particles. The average particle size histogram of BS S was found to be 13.58 nm (Figure S2a). TEM analysis of SPIONs demonstrated the presence of spherical-shaped agglomerated particles (Figure 2d). The variation in size between SPIONs and BS-S suggested that the increase in size could be attributed to the conjugation of the drug [31]. The monodispersity of BS-S indicated the stability of SPIONs after their conjugation with β-sitosterol. BS-SP Figure 2b was observed to be agglomerated with an increase in size, whereas BS-SPP demonstrated the presence of spherical particles, monodispersed particles resembling the morphology observed with BS-S. The increase in size of the BS-SP and BS-SPP can be attributed to the polymer coating. The average particle size histogram for BS-SP and BS-SPP was found to be 29.62 nm and 8.65 nm, as shown in the Supplementary Data (Figure S2b,c). These findings align with a previous study by Shamim and his coworkers [32], where the size of polymer (PNIPAM) coated magnetic nanoparticles was observed to be larger compared to their uncoated counterparts. The resulting conjugated complex, BS-SPP, exhibited monodispersed conjugates; notably, the morphology of BS-SPP differed from that of BS-S and BS-SP. The BS-SPP complex exhibited a distinctive stripe-like structure, with sizes ranging from 25 ± 10 nm. The observed distance between two stripe-like structures was approximately 0.25 nm, as illustrated in Figure 2d. The surface morphology of BS-S and BS-SPP was analyzed with SEM. The surface of BS-SPP was observed to be smooth with a white cloudy appearance on the surface, further confirming the conjugation of polymers (Figure S3a,b).

### 3.2. In Vitro Adsorption of BS-S, BS-SP, and BS-SPP

**Figure 3:**
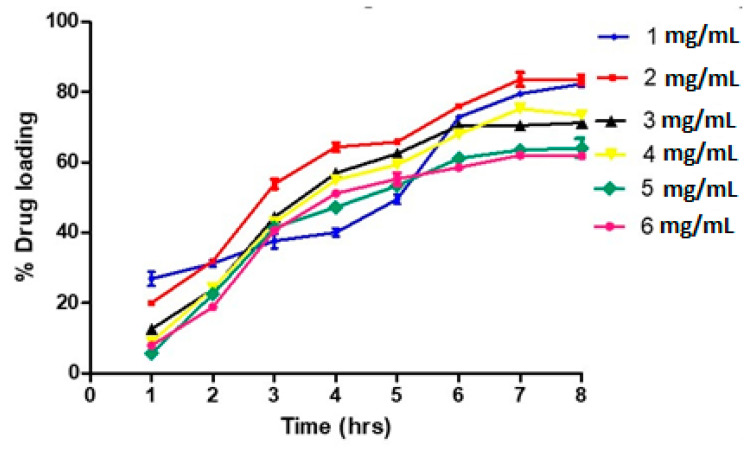
Drug encapsulation efficiency observed with SPIONs.

Various concentrations of BS ranging from 1 mg/mL to 6 mg/mL were loaded onto 5 mg of SPIONs, and the maximum drug loading was achieved with 5 mg/mL and 6 mg/mL with a drug encapsulation efficiency of 61%, as shown in Figure 3.

**Figure 4:**
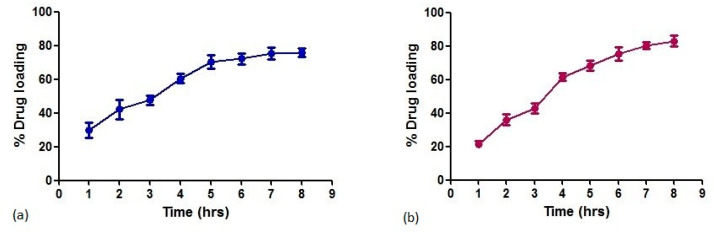
(**a**,**b**) Represent the drug encapsulation efficiency observed with BS-SP and BS-SPP.

Hence, the concentration of 5 mg/mL of BS was chosen for further experiments. Since maximum drug adsorption was achieved at a 5 mg/mL concentration of β-sitosterol, the same concentration was used with BS-SP. About 74.9% of drug encapsulation efficiency was achieved with BS-SP, as shown in Figure 4a. The increase in drug-loading capacity indicates the role of polymer in drug entrapment with the SPIONs. SPIONs conjugated with PEG and PNIPAM showed an increased drug encapsulation efficiency of 82.5% (Figure 4b).

This shows that the addition of two polymers can effectively entrap the drug compared to single polymer PNIPAM. The conjugation of two polymers increased the drug loading and encapsulation efficiency of drugs [32]. Further, the use of two polymers increases the loading of hydrophobic drugs effectively and avoids RES uptake with increased blood circulation time [33].

### 3.3. In Vitro Release Profile of BS-S, BS-SP, and BS-SPP

The drug release behavior of BS-S, BS-SP, and BS-SPP was evaluated in phosphate saline buffer [34,35].

**Figure 5:**
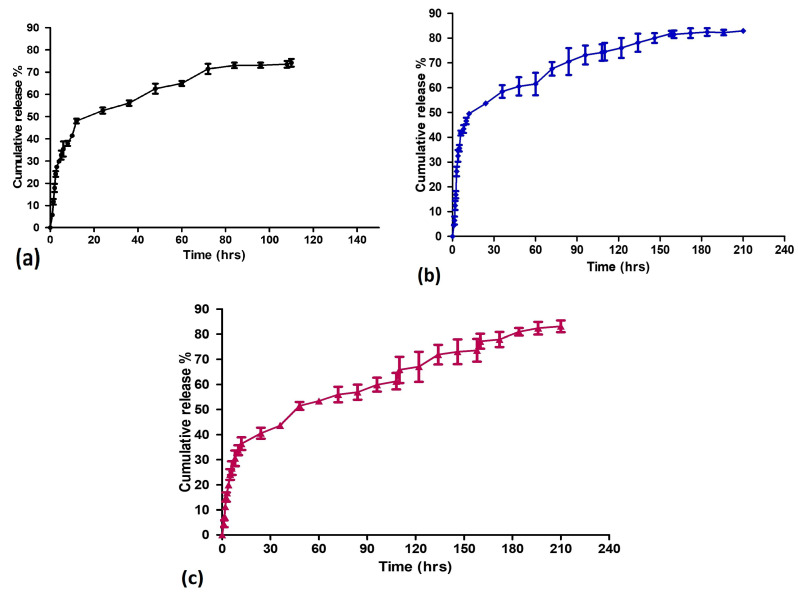
The drug release profile of (**a**) BS-S, (**b**) BS-SP, and (**c**) BS-SPP.

The results, as depicted in Figure 5a, revealed that approximately 74% of the loaded BS was released from the SPIONs after 110 h. The release profile exhibited an initial burst effect, with 40% of the drug being released within the first 6 h. This initial burst release can be attributed to the rapid release of the drug that was loosely bound to the SPIONs. In BS-SP and BS-SPP, an initial burst release was observed within 12 h, with 35.5% of drug release in BS-SPP when compared to that of BS-SP (49%). Despite the initial rapid release, sustained drug release was achieved in both BS-SP and BS-SPP formulations, with maximum drug releases of 82.6% and 83%, respectively. In both BS-SP (Figure 5b) and BS-SPP (Figure 5c) formulations, the amount of drug release remained constant from 84 to 210 h.

### 3.4. Impact of BS-S, BS-SP, and BS-SPP on Cancer Cell Viability

**Figure 6:**
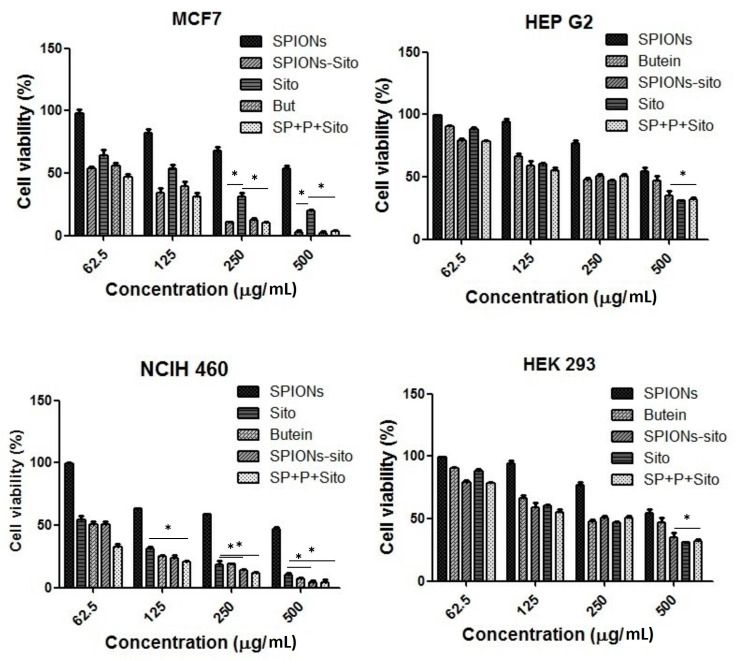
In vitro cytotoxic assay of BS conjugated with SPIONs and polymers towards MCF7, HEP G2, NCIH 460, and HEK 293 cells. * *p* < 0.05.

MCF7, HEPG2, and NCIH460 cancer cells, as well as kidney cells (HEK293) expressing EGFR and MET receptors, were subjected to varying concentrations (62.5, 125, 250, and 500 µg/mL) of SPIONs, BS-S, BS-SPP, and Butein to predict its cytotoxic effect by MTT dye reduction assay [36]. The IC50 value obtained for BS, BS-S, BS-SP and BS-SPP with their control butein are shown in Table 2. Figure 6 demonstrates the dose–response relationship between the metabolic activity of EGFR and MET receptor-expressing cells and different concentrations of SPIONs, BS-S, BS-SP, and BS-SPP. A significant difference was not observed with uncoated SPIONs, except at a higher concentration of 500 µg/mL, suggesting that they exhibit anticancer activity only at higher concentrations in each cell. Concentration-dependent anticancer effects were observed for BS-S, BS-SP, and BS-SPP in all cancer cell lines.

The IC50 value of BS-S toward MCF7, HEPG2, and NCIH460 cancer cells were 197, 261, and 185 µg/mL, respectively. Similarly, the effective inhibitory concentrations (IC50) for BS-SP and BS-SPP against these cells were found to be 168 µg/mL, 233 µg/mL, 175 µg/mL, and 149 µg/mL, 207 µg/mL, 164 µg/mL, respectively.

The results indicate that polymeric-conjugated SPIONs with BS exhibited higher anticancer activity compared to SPIONs with BS alone. The increased anticancer activity observed may be attributed to enhanced drug accumulation and sustained drug release facilitated by the action of polymers [37,38]. The IC50 sigmoidal curves for cell viability assay in each cell line are shown in the Supplementary Data (Figures S4–S7). Maximum IC50 values of BS-SP were observed in breast cancer cells, followed by NSCLS cells. However, significant inhibitory activity was observed in the NSCLC cells for all tested samples, including BS-S, BS-SP, and BS-SPP. Thus, the designed drug complex shows promise as a potent candidate for both NSCLC and breast cancer treatment.

### 3.5. BS-SPP Downregulate Expression of Both EGFR and MET Receptor in NCIH460 Cells

**Figure 7:**
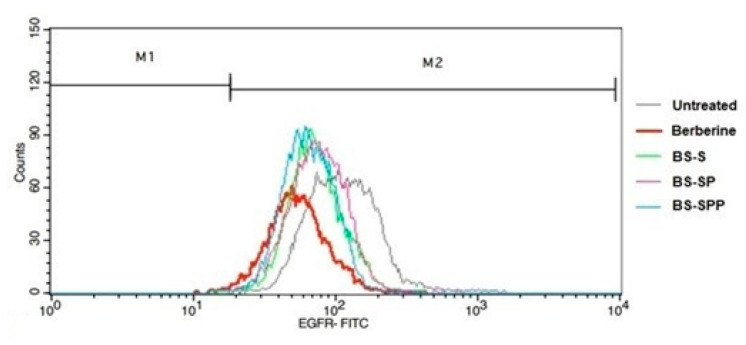
Expression of EGFR in berberine, BS-S, BS-SP, and BS-SPP treated NCIH460 cells.

To validate the inhibitory effects of BS-S, BS-SP, and BS-SPP on the protein expression levels of EGFR and MET receptors, flow cytometric analysis was conducted [39,40]. In the cell viability studies, it was evident that BS-SPP demonstrated cytotoxic activity in NSCLC cells characterized by elevated levels of both EGFR and MET receptors. NCIH460 cells were treated with BS-S, BS-SP, and BS-SPP at their effective inhibitory concentrations, 185 µg/mL, 175 µg/mL, and 164 µg/mL, respectively, for 24 h. The histogram obtained from flow cytometric analysis reveals lower expression of EGFR and MET receptors in NCIH460 cells in BS-S, BS-SP, and BS-SPP treated cells. Further analysis revealed that the downregulation of EGFR was more evident in BS-SPP-treated cells compared to those treated with BS-S and BS-SP (Figure 7). These findings indicate that maximum suppression of EGFR expression was achieved using BS-SPP conjugated complex stabilized using both PEG and PNIPAM-NH2 polymers [15]. These results provide additional evidence for the inhibitory effects of the drug complexes on EGFR and MET receptor expression levels.

**Figure 8:**
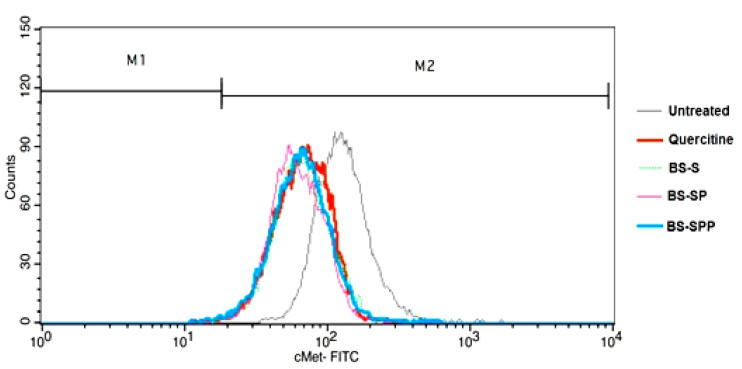
Expression of MET receptor in berberine, BS-S, BS-SP, and BS-SPP treated NCIH460 cells.

The expression levels of MET receptors in NCIH460 cells were observed to be similar in BS-SP and quercetin (control drug) treated cells [41]. However, the downregulation of the MET receptor was higher in BS-SP compared to BS-SPP and BS-S (Figure 8). Flow cytometric analysis revealed that the SPION-PNIPAM conjugated complex (BS-SP) induced a greater increase in cell death through apoptosis than the complex involving SPION conjugated with PEG and PNIPAM polymers (BS-SPP). Both BS-SP and BS-SPP demonstrated the ability to induce apoptosis through the downregulation of the MET receptor.

These findings suggest that BS-SP has a stronger impact on reducing MET receptor expression in NCIH460 cells, indicating its potential as a therapeutic approach. Further optimization of the concentration ratios between PEG and PNIPAM or between SPION and polymers may enhance the efficacy of MET receptor downregulation in BS-SPP-treated NCIH460 cells.

## 4. Discussion

In this study, we evaluated the increase in the efficiency of BS by adding SPIONs and biodegradable polymers. EGFR and MET receptor cross-talk bypass targeted therapies, promote cell motility and invasiveness, and activate survival pathways, leading to treatment resistance and poorer prognosis. Despite the extensive research and ongoing studies, inhibiting the cross-talk between the EGFR and MET signaling pathways in cancer remains a challenge. BS is a well-known plant bioactive molecule that possesses anticancer activity towards various cancer cell lines. Hence, by targeting EGFR-MET cross-talk inhibition with BS, we hold promise for combating tumor progression and metastasis. To our knowledge, this is the first report on targeting EGFR and MET receptor inhibition with BS conjugated with biodegradable polymers.

BS was proven to exhibit promising anticancer activity in various cancer cell lines. However, the hydrophobic nature of the drug poses limitations to its efficacy. In our study, we aimed to overcome this challenge by enhancing the activity of BS through conjugation with SPIONs and biodegradable polymers to improve the solubility and bioavailability of the drug, ultimately enhancing its therapeutic efficacy against various cancer cell lines. To comprehensively evaluate the efficacy of BS, we conducted a series of experiments involving its conjugation with SPIONs, SPIONs combined with PEG, and SPIONs-PEG-PNIPAM complex. PEG is commonly used in drug delivery systems due to its ability to improve the stability, biocompatibility, and circulation time of nanoparticles [42]. PEGylation, which involves the attachment of PEG chains to the surface of nanoparticles, can increase their hydrodynamic size and provide steric stabilization, reducing their clearance by the reticuloendothelial system [43]. Similarly, the incorporation of PNIPAM, a thermoresponsive polymer, can also affect the size of the nanoparticle drug complex [44]. The combination of PEG and PNIPAM with BS and SPIONs can create a synergistic effect, contributing to the overall increase in size. The hydrophilic PEG chains provide a hydration layer around the nanoparticles, while the temperature-responsive PNIPAM can contribute to the overall size modulation. This size increase has implications for drug delivery, as it affects important parameters such as drug loading capacity, circulation time, and interactions with biological systems. The objective of this work was to elucidate the role of each molecule in enhancing the efficiency of BS.

BS was successfully conjugated with SPIONs, SPIONS-PNIPAM, and SPIONS-PEG-PNIPAM. The increase in mean hydrodynamic size and stability of the BS-S, BS-SP, and BS-SPP are shown in the table. Consecutive increase in the hydrodynamic size of BS-SP and BS-SPP when compared to BS-S suggests the successful conjugation of BS with the SPIONs and polymers. The addition of polymeric agents such as PEG and PNIPAM in nanoparticle drug complexes is known to have an impact on their size. The observed increase in size is consistent with previous studies that have reported similar size increases upon conjugation of drugs or ligands with nanoparticles [41,45]. The stability of the conjugated complex was assessed using zeta potential analysis. The results showed a significant increase in stability from −15.8 mV for SPIONs to −48.7 mV for BS-S (Figure S1a,b). The higher magnitude of the zeta potential indicates a stronger electrostatic repulsion between the nanoparticles, which contributes to their enhanced stability [46].

The increase in stability upon conjugation of BS can be attributed to the presence of functional groups on BS that interact with the surface of SPIONs, thereby reducing particle aggregation and enhancing stability [47], and increasing drug delivery [48]. Higher stability was achieved with BS-SPP (−62.4 mV) compared to BS-SP (−54.2 mV) and BS-S (−48.7 mV), indicating the increase in stability due to the addition of two polymers. The incorporation of PEG can create a repulsive layer around the nanoparticles, leading to an increased zeta potential and improved stability [49]. Similarly, PNIPAM-NH_2_ being a charged polymer, can contribute to the electrostatic stabilization of the complex, resulting in higher zeta potential. The larger hydrodynamic size of the BS-SPP can facilitate the passive targeting of the nanoparticles to tumor tissues through the enhanced permeation and retention effect (EPR) [42]. Additionally, the increased stability of the complex prevents oxidation or degradation of the magnetic nanoparticles during storage or circulation in the bloodstream [50]. This enhanced stability ensures the integrity of the SPION complex, leading to improved drug delivery efficiency and therapeutic efficacy.

TEM analysis of SPIONs, BS-S, BS-SP, and BS-SPP variations in size and dispersity of the particles. SPIONs exhibited agglomeration and had a size of 10 ± 20 nm, as shown in Figure 2b. BS-S exhibited a narrow size distribution with monodispersed particles with a size of 20 ± 10 nm, whereas BS-SP and BS-SPP were in the size range of 25 ± 10 nm, as depicted in Figure 2a. This uniform size distribution is desirable for drug delivery systems as it ensures consistent drug loading and release [51]. The monodispersity of the particles can be attributed to the conjugated complex, which effectively controlled the attachment of BS molecules onto the SPIONs’ surface [52]. Previous studies have reported an increase in size of approximately 50 nm upon the conjugation of magnetic nanoparticles with chitosan-coated nanoparticles [53]. However, in the case of BS-S, no significant difference in size was observed after β-sitosterol conjugation. This may be due to the presence of a single-layer coating of the drug or the minimal space occupied by the drug molecules, preventing a substantial increase in the size of the conjugated complex.

An increase in the size of BS-SP and BS-SPP is due to the coating of polymeric agents. This increase in size observed in TEM analysis corresponds well with the DLS data SEM analysis reveals smooth surface morphology of BS-SPIONs compared to SPIONs indicating the presence of polymer on the surface of SPIONs, confirming the conjugation of polymer onto SPIONs. Further, nanoparticles with sizes below 100 nm have demonstrated efficient evasion of the EPR effect, thereby enabling improved tissue penetration and targeted drug delivery [42,54]. This indicates that the conjugated complexes, which fall within this size range, hold the potential for enhanced therapeutic efficacy.

The drug encapsulation efficiency is a crucial parameter in drug delivery systems as it determines the amount of drug effectively encapsulated within the nanoparticles. High encapsulation efficiency ensures that a significant proportion of the drug is available for therapeutic action, reducing wastage and maximizing the therapeutic efficacy of the treatment. Comparing the drug encapsulation efficiency and loading capacity of β-sitosterol with other anticancer drugs and biomolecules, it is evident that β-sitosterol performs competitively. For instance, the anticancer drug 5-fluorouracil demonstrated a drug encapsulation efficiency of 64% and a drug loading capacity of 45% [22]. Similarly, the biomolecule curcumin exhibited a drug encapsulation efficiency of 44.4% [55].

About 74.9% of drug encapsulation efficiency was achieved with BS-SP, as shown in Figure 3. This indicates that the SP polymer alone was able to effectively entrap a significant portion of the drug within the SPIONs. However, drug encapsulation efficiency was further enhanced by conjugation with additional polymer PNIPAM. The results showed a notable improvement, with an increased drug encapsulation efficiency of 82.6% when using the SPIONs conjugated with PEG and PNIPAM. SPIONs conjugated with PEG and PNIPAM showed an increased drug encapsulation efficiency of 82.5% (Figure 4b). This shows that the addition of two polymers can effectively entrap the drug compared to single polymer PNIPAM. This was consistent with the earlier report [32]. Further, the use of two polymers increases the loading of hydrophobic drugs effectively and avoids RES uptake with increased blood circulation time [33].

The drug release profile of BS in conjugation with SPIONs and polymers is shown in Figure 5a–c. Approximately 74% of the loaded β-sitosterol was released from the SPIONs over a duration of 110 h with 40% of initial burst effect. However, a sustained release of the drug was achieved over a period of 100 h, which is comparable to the release profile of doxorubicin-conjugated SPIONs [56] released at 70 h. This comparison implies that the release duration of β-sitosterol is similar to that of Doxorubicin when conjugated with SPIONs. Hence, this suggests that the β-sitosterol-loaded SPIONs have the potential for sustained drug delivery, similar to the established drug doxorubicin.

The initial burst was found to be less in BS-SP and BS-SPP, indicating better drug encapsulation and strong interaction of the drug with the SPION-polymeric complex. The observed result was similar to other drugs loaded with magnetic nanoparticles [57]. Despite the initial burst release, sustained drug release was achieved in both BS-SP and BS-SPP complexes, with approximately 83% of the drug being released from both conjugated complexes. Although the initial burst was different, the long-term release profiles of the drug were comparable for both SP and SPP. The sustained drug release could be attributed to the controlled release mechanism provided by both the SPION-polymeric complex. Previous studies have shown that the addition of hydrophilic polymers such as polyethylene glycol (PEG) and polyethyleneimine (PEI) can prolong drug release, likely due to the presence of polymeric chains that impede the entry of water into the complex [35].

BS-S, BS-SP, and BS-SPP, exhibited concentration-dependent anticancer activity in each cancer cell line with high expression of EGFR and MET receptors. Among the conjugated complexes, BS-S showed a maximum IC50 value in all the cell lines, indicating its lower potency for drug delivery applications. The incorporation of polymers enhanced the bioavailability of the drug at the intended target site, showing greater activity. The incorporation of polymers enhances the bioavailability of the drug at the intended target site [58]. Activity with BS-SP and BS-SPP in all the cancer cell lines compared to BS-S. Further, the combination of SPIONs with PEG and PNIPAM in the designed drug complex may have resulted in synergistic effects, leading to improved inhibitory activity against all the cell lines. When comparing the effectiveness of two different polymeric conjugations, viz., PEG and PEG-PINIPAM (SP and SPP), SPP expressed lower IC50 values in the MCF7, HEPG2, and NCIH460 cell lines, indicating greater potency and effectiveness in inhibiting the cell growth with a lower concentration of the drug conjugated to each complex. However, superior inhibitory activity with the lowest IC50 was observed in NSCLC cells with all the tested samples. Hence, the designed drug complex could be used as a potent drug candidate for NSCLC.

To gain further insights into the effects of BS along with its conjugated complex on NSCLC, flow cytometric analysis was conducted. The results of the cell viability assay were found to be well correlated with the flow cytometric analysis. Specifically, the BS-SPP conjugated complex demonstrated higher cytotoxic activity against NSCLC cells that expressed elevated levels of both the EGFR and MET receptors. The expression level of EGFR in BS-SP and BS-S were almost similar, with only a 0.6% increase in the apoptotic stage of NSCLC cells treated with BS-SP. The expression of EGFR was also much lower in BS-SP-treated cells compared to the control drug berberine [59], with BS-SP showing a 14% increase in suppression of EGFR. The expression levels of MET receptors in NCIH460 cells were observed to be similar in BS-SP and quercetin [29] (control drug) treated cells. This indicates that both BS-SP and quercetin had comparable effects on the expression of the MET receptor. However, it was found that BS-SP exhibited a higher downregulation of the MET receptor compared to BS-SPP. However, both BS-SP and BS-SPP induced apoptosis through the downregulation of the MET receptor.

## Figures and Tables

**Table 1:** Hydrodynamic size and zeta potential of SPIONs and polymer conjugates.

Nanoparticle	DLS Mean (nm)	Polydispersity INDEX (PI)	Standard Deviation (SD)	Zeta (mV)
SPIONs	90.3	0.373	14.4	−15.8
BS-S	113	0	4.2	48.7
BS-SP	162.3	0	4.8	−54.2
BS-SPP	381.8	0	54.3	−64.2

**Table 2:** The IC50 value of BS-S, BS-SP, and BS-SPP.

Cancer Cell Lines	IC50 Value (µg/mL) of BS-S	IC50 Value (µg/mL) of BS-SP	IC50 Value (µg/mL) of BS-SPP	IC50 Value (µg/mL) of BS	IC50 Value (µg/mL) of Butein
MCF7	197	168	149	242	163
HEPG2	261	233	207	274	298
NCIH460	185	175	164	191	178

## Data Availability

Not applicable.

## Supplementary Materials

The following supporting information can be downloaded at: https://www.mdpi.com/article/10.3390/pharmaceutics15082158/s1, Figure S1: Zeta potential of SPIONs, BS-S, BS-SP and BS-SPP. Figure S2: Average particle size histogram of SPIONs, BS-S, BS-SP and BS-SPP. Figure S3: Surface topographical images illustrating the change in the morphology between (a) BS-S and (b) BS-SPP. Figures S4–S7: IC50 sigmoidal curves for HEK 293, HEP G2, MCF7 and NCIH 460 cell lines.
