# Supplementary material for: Modulating the Effect of β-Sitosterol Conjugated with Magnetic Nanocarriers to Inhibit EGFR and Met Receptor Cross Talk
Source: Pharmaceutics. 2023 Aug 19;15(8):2158. doi: 10.3390/pharmaceutics15082158 (PMC10458314; doi:10.3390/pharmaceutics15082158)
Supplement: Supplementary file 1 [file pharmaceutics-15-02158-s001.zip › pharmaceutics-2509951-supplementary.pdf]

Modulating the effect of β-Sitosterol conjugated with magnetic nanocarriers to inhibit EGFR and Met receptor cross talk

Shanmuga Sundari Ilangovan[1], Biswanath Mahanty[2], Venkatesan Perumal[3], Shampa Sen[4$*]

1 Department of Biotechnology, Bannari Amman Institute of Technology, Sathyamangalam, India; shanmugasundarii@bitsathy.ac.in
2 Department of Biotechnology, Karunya Institute of Technology and Sciences, Coimbatore, India; bmahanty@gmail.com
3 Center for Injury Biomechanics, Materials and Medicine, Department of Biomedical Engineering, New Jersey Institute of Technology, Newark, NJ 07102, USA; perumal@njit.edu
* School of Biosciences and Technology, VIT, Vellore, India; shampa.vitu@gmail.com
$ Edumatter, Larica Township, Barrackpore road, Barasat, Kolkata-700126, West Bengal, India

Corresponding author: Dr. Shampa Sen

Co-corresponding author: Dr. I. Shanmuga Sundari

Experimental results:

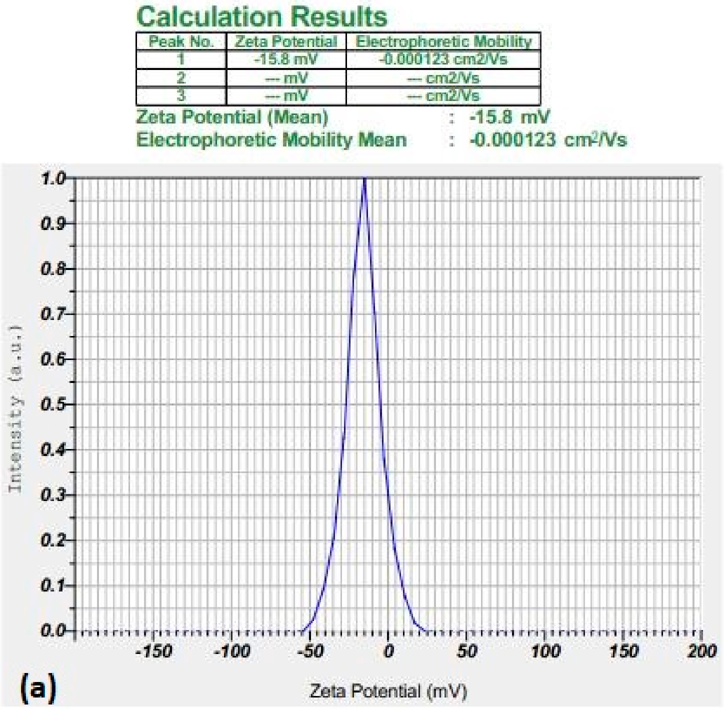

Calculation Results

Peak No.	Zeta Potential	Electrophoretic Mobility
1	-48.7 mV	-0.000378 cm2/Vs
2	--- mV	--- cm2/Vs
3	--- mV	--- cm2/Vs

Zeta Potential (Mean) : -48.7 mV
Electrophoretic Mobility Mean : -0.000378 cm^2/Vs

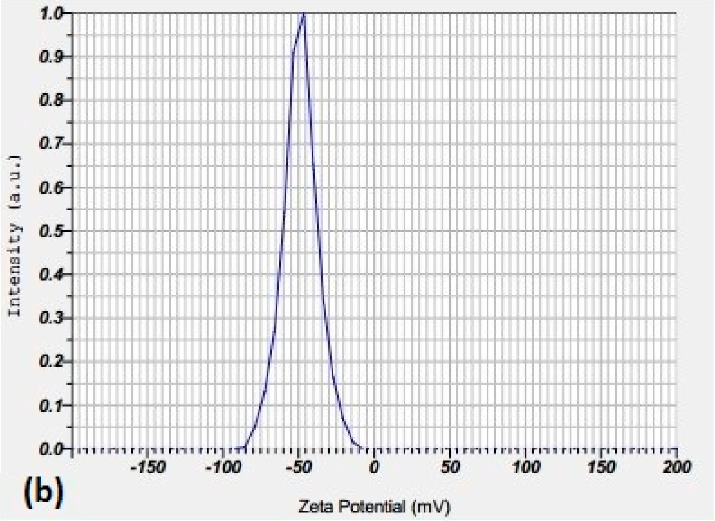

(b)

Calculation Results

Peak No.	Zeta Potential	Electrophoretic Mobility
1	-54.2 mV	-0.000421 cm2/Vs
2	--- mV	--- cm2/Vs
3	--- mV	--- cm2/Vs

Zeta Potential (Mean) : -54.2 mV
Electrophoretic Mobility Mean : -0.000421 cm^2/Vs

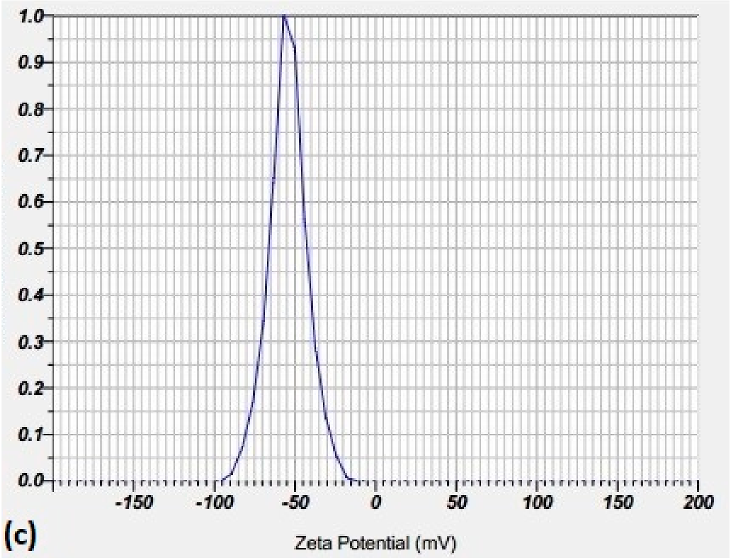

(c)

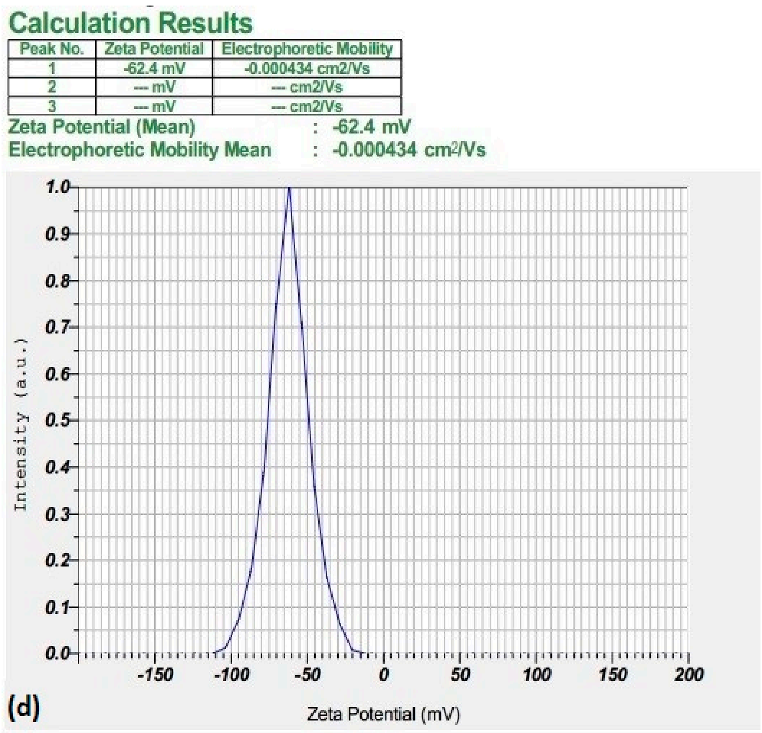

Figure S1. (a), (b), (c) and (d) represents the zeta potential of SPIONs, BS-S, BS-SP and BS-SPP

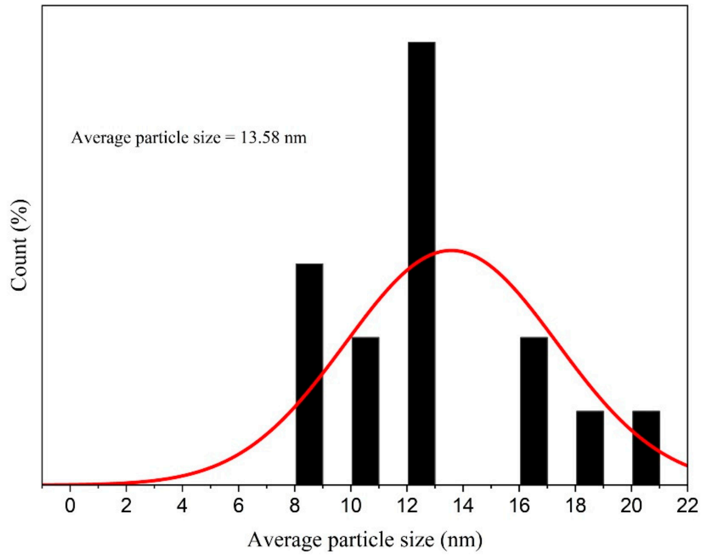

Figure S2. (a) Average particle size histogram of BS S

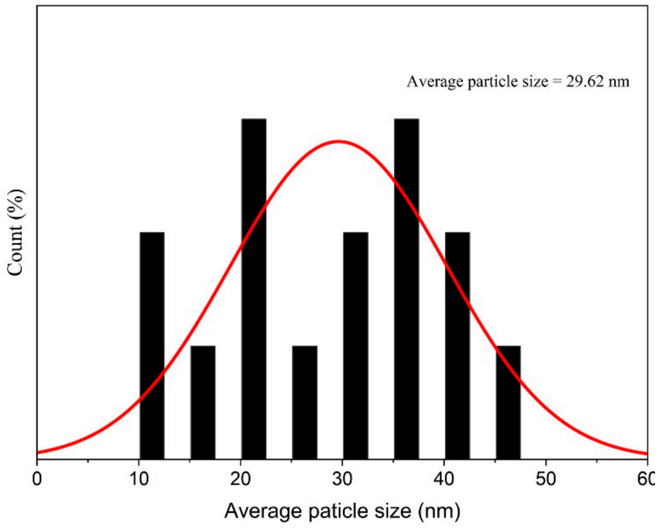

Figure S2. (b) Average particle size histogram of BS SP

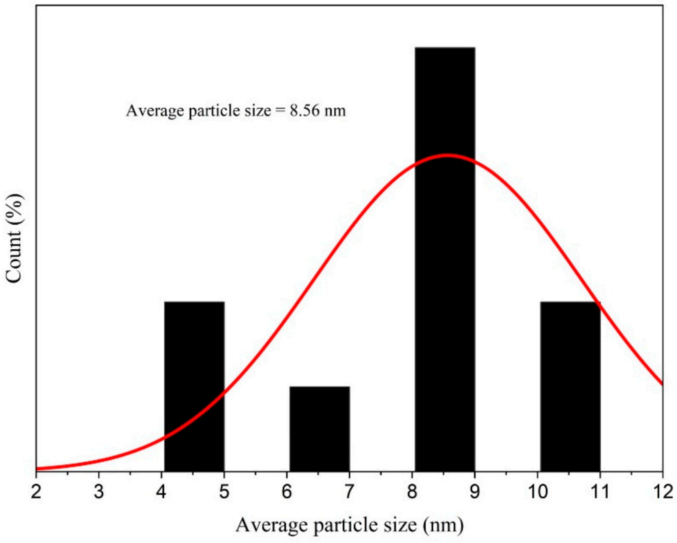

Figure S2. (c) Average particle size histogram of BS SPP

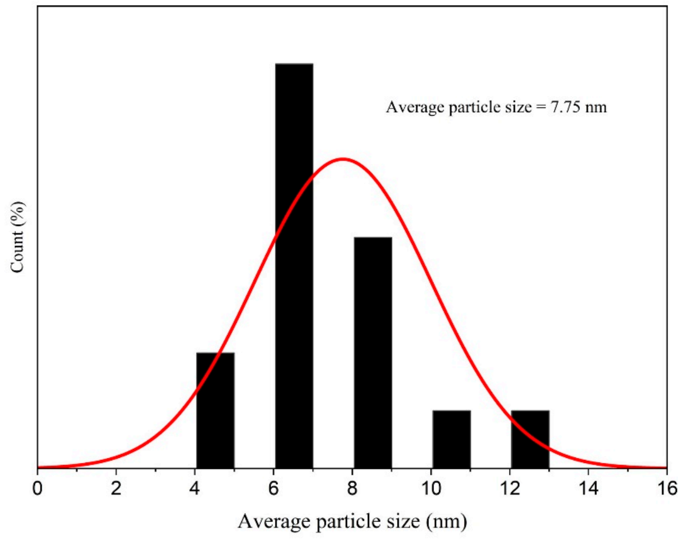

Figure S2. (d) Average particle size histogram of SPIONs

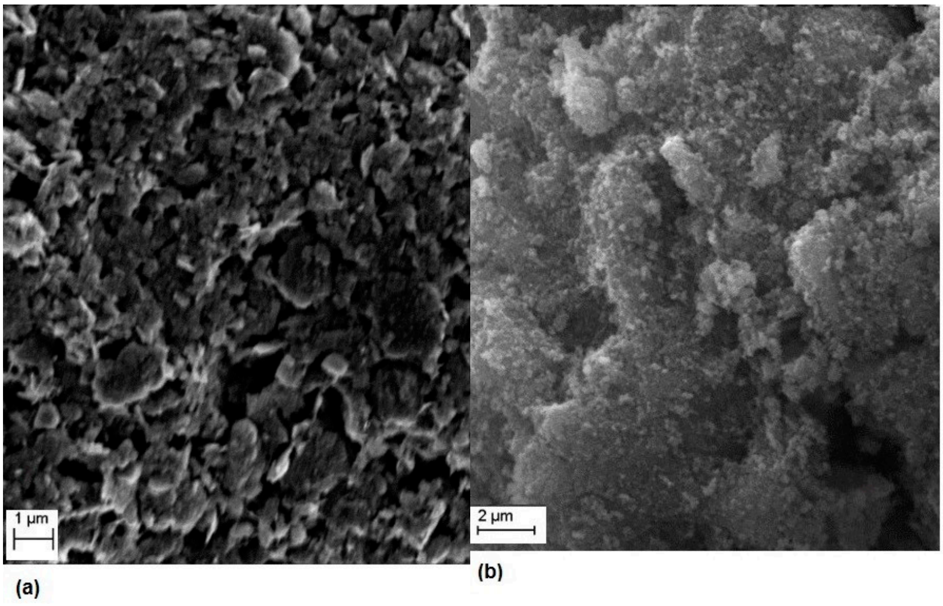

Figure S3. Surface topographical images illustrating the change in the morphology between (a) BS-S and (b) BS-SPP

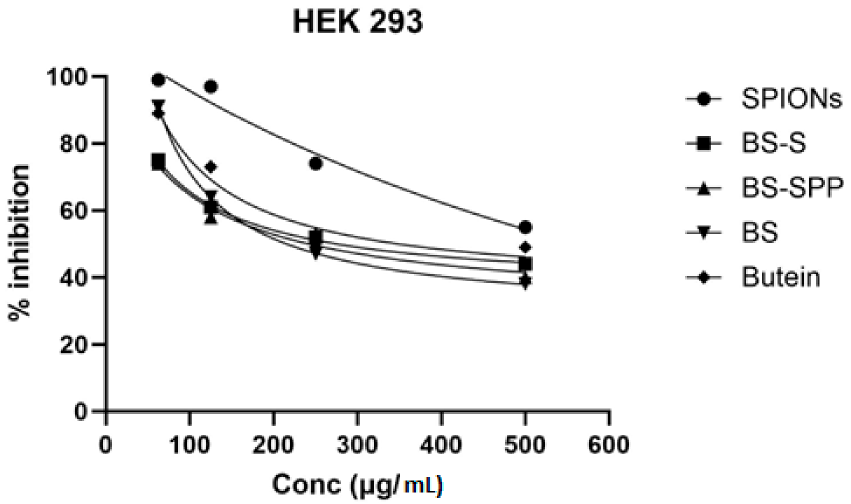

Figure S4. IC50 sigmoidal curves for HEK 293 cell line

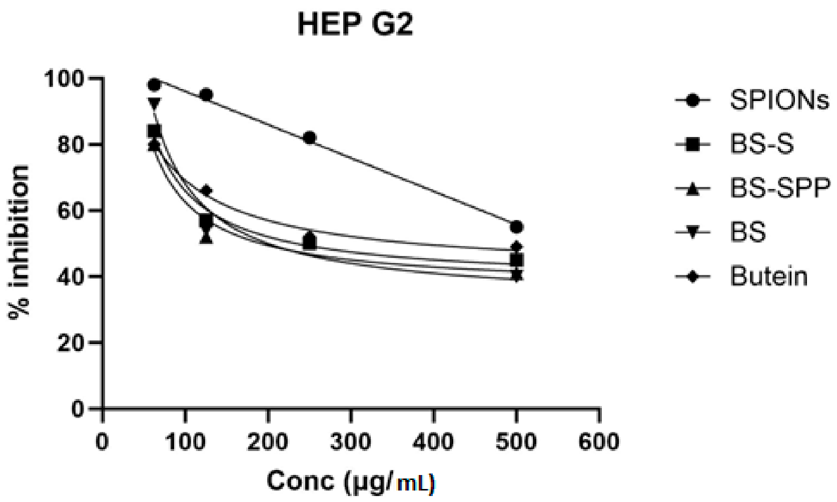

Figure S5. IC50 sigmoidal curves for HEP G2 cell line

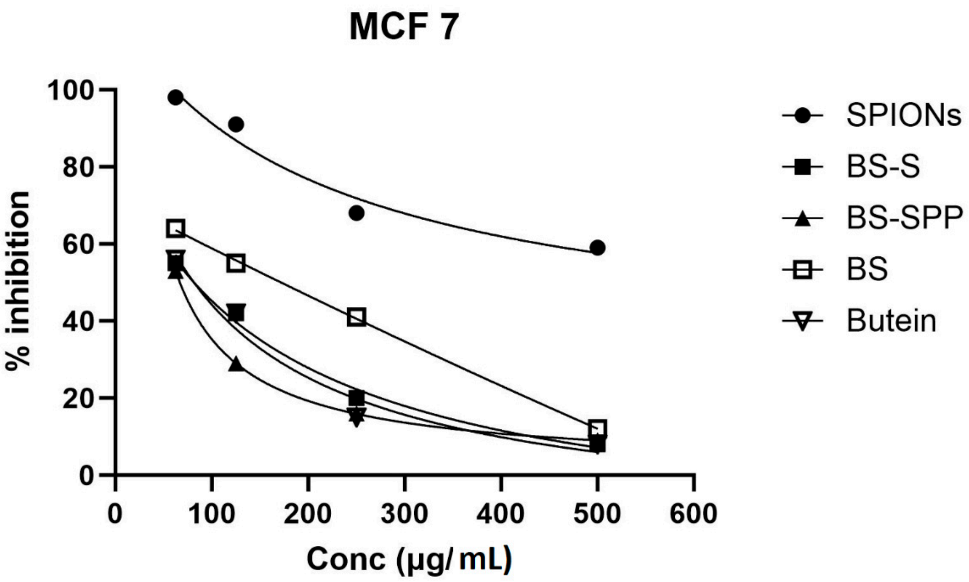

Figure S6. IC50 sigmoidal curves for MCF7 cell line

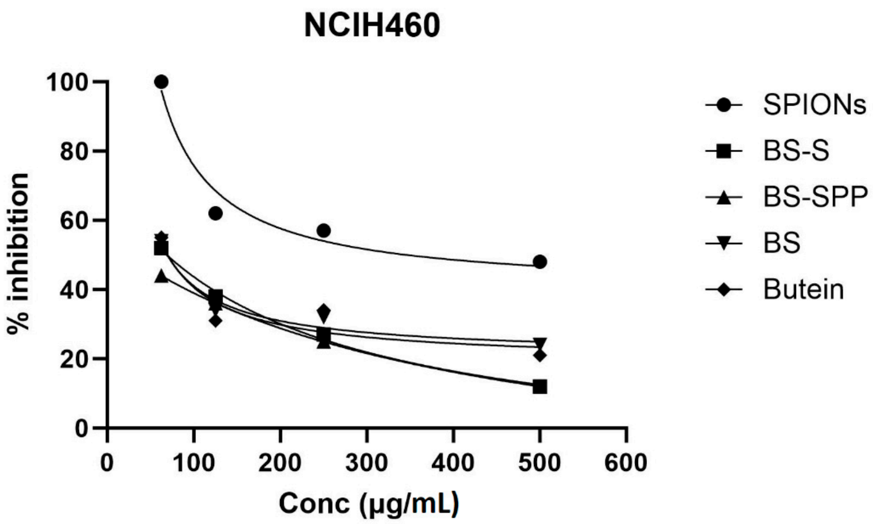

Figure S7. IC50 sigmoidal curves for NCIH 460 cell line
